# Pacemaker Lead Perforation Presenting as Persistent Abdominal Pain: A Case Report

**DOI:** 10.7759/cureus.84116

**Published:** 2025-05-14

**Authors:** Drew W Barron, Nathan G Rasmussen, Mark H Auerbach, Douglas Rappaport, Wayne A Martini

**Affiliations:** 1 Emergency Medicine, Mayo Clinic Alix School of Medicine, Scottsdale, USA; 2 Emergency Medicine, Mayo Clinic Arizona, Phoenix, USA

**Keywords:** cardiac device complications, computed tomography, myocardial perforation, pacemaker lead perforation, pacemaker migration, persistent abdominal pain

## Abstract

Cardiac pacemakers are widely used, and lead perforation is a rare but serious complication that may present atypically, leading to diagnostic challenges. We present the case of a 79-year-old male with a history of multiple comorbidities, including heart failure with preserved ejection fraction, right bundle branch block, and recent dual-chamber pacemaker implantation, who developed persistent left upper quadrant abdominal pain. Despite multiple ED visits and specialist evaluations, no clear etiology was identified. Previous outside hospital diagnostic workups, including electrocardiography, chest radiography, and CT, failed to reveal an acute cause for his symptoms. Ultimately, CT imaging in our ED demonstrated migration of the right atrial pacemaker lead, with positioning suggestive of myocardial perforation. The patient was admitted for further management, remained hemodynamically stable with pain controlled, and was conservatively monitored without immediate intervention.

Myocardial perforation due to pacemaker leads can occur acutely, subacutely, or as a delayed complication. While early perforation may be identified during implantation or shortly thereafter, subacute and chronic cases often present with non-specific symptoms, making diagnosis challenging. Imaging modalities such as CXR, echocardiography, and CT play a critical role in identifying lead migration. Management strategies range from conservative observation in stable patients to surgical or transvenous lead extraction in symptomatic or high-risk cases.

This case stresses the importance of considering pacemaker-lead migration as a potential etiology in patients presenting with unexplained pain after implantation. It also highlights the limitations of conventional diagnostic tools and the value of advanced imaging techniques, such as CT, in the timely identification of this complication.

## Introduction

Cardiac pacemakers are common medical devices. It is estimated that over 1,000,000 cardiac pacemakers are implanted every year, a number which has been growing for the last several decades [1,2]. While complications of cardiac pacemaker implantation are rare, the prevalence of pacemakers means that healthcare providers will likely encounter them. These complications can include infection, lead dislodgement, and cardiac perforation, among others [3,4].

Cardiac perforation is a particularly rare complication of cardiac pacemaker implantation, with an estimated incidence of 0.5%-2%, according to reports [5-7], with numerous cases reported [8-11]. Symptoms of cardiac perforation may include chest pain, dyspnea, syncope, or manifestations of phrenic nerve stimulation or irritation like hiccups or abdominal spasms [5,8-12]. Due to the wide variability in symptom presentation, pacemaker lead perforation remains a diagnostic challenge. This case highlights the limitations of conventional diagnostic tools and the necessity of considering lead migration in patients with persistent, unexplained pain after implantation.

## Case presentation

We present a 79-year-old male who developed left-sided parasternal and left upper quadrant abdominal pain following implantation of a cardiac pacemaker and who was evaluated multiple times in EDs over the course of several months before presenting to our ED for evaluation.

The patient has a medical history notable for hypertension, type 2 diabetes mellitus, hyperlipidemia, chronic obstructive pulmonary disease, heart failure with preserved ejection fraction, right bundle branch block, laryngeal cancer status after radiation, and an infrarenal abdominal aortic aneurysm, right common iliac artery aneurysm, and acute renal failure requiring intermittent hemodialysis. His surgical history includes a dual-chamber pacemaker system insertion for a complete heart block 16 weeks before, with right atrial lead dislodgement and revision to presentation, and an open hernia repair. The patient presented to the ED with a chief concern of persistent left upper quadrant abdominal pain for the past seven weeks.

The patient experienced persistent left upper quadrant abdominal pain for seven weeks, leading to multiple evaluations. His cardiologist assessed him several times but did not attribute the pain to his pacemaker. He presented to an outside ED three times before evaluation in our ED. Six weeks before, he underwent laboratory testing, EKG, CXR, and CT of the abdomen and pelvis, which revealed influenza and incidental findings of a 4.3 cm infrarenal abdominal aortic aneurysm and a 2.3 cm right common iliac artery aneurysm; he was discharged home. Three weeks before, he was noted to have left upper quadrant abdominal distension and tenderness without guarding. Further evaluation, including labs, EKG, and CT of the abdomen and pelvis, showed no acute findings, and a representative confirmed his pacemaker to be functioning properly. A slightly elevated D-dimer with low pretest probability ruled out pulmonary embolism, and he was discharged with a diagnosis of left rib cage tenderness after a short observation period. One week prior, he returned with persistent left upper quadrant pain and tenderness in the left upper quadrant but was again discharged after negative labs, EKG, CXR, and CT of the chest and abdomen. He was advised to follow up with a vascular surgeon and a gastrointestinal (GI) specialist as an outpatient.

Upon arrival, the patient reported persistent left upper quadrant abdominal pain, rated 8/10 in severity, with a waxing and waning course, consistent with previous evaluations. He denied any modifying factors, noting that the pain was not influenced by food, movement, or breathing. However, he described a tingling sensation radiating to the left side of his neck. He also denied recent fever, chills, rash, appetite changes, nausea, vomiting, diarrhea, constipation, hematochezia, dysuria, urinary frequency, urinary urgency, neck pain, back pain, weakness, seizures, or headaches.

On initial evaluation, the patient appeared non-toxic and in no acute distress. He was hypertensive, with a blood pressure of 166/93 mmHg, while his other vital signs were within normal limits. Breath sounds were clear to auscultation bilaterally, with a regular respiratory rate and unlabored effort. The abdominal examination was benign, revealing normal bowel sounds, no distension, tenderness, rebound, or guarding. The remainder of his physical exam was unremarkable.

**Figure 1:**
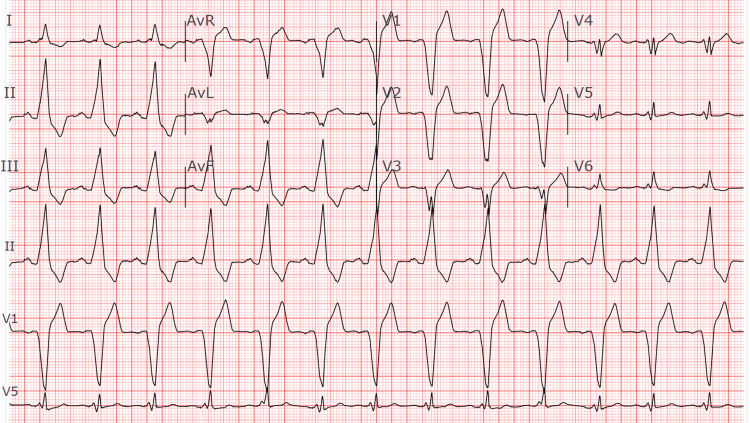
Electrocardiogram showing atrial-sensed ventricular-paced rhythm with a heart rate of 72 beats per minute.

Given his seven-week course of persistent pain out of proportion to findings, multiple previous visits, and past imaging without identifying a root cause of his pain, the differential diagnosis was broad. Possibilities included a GI etiology, for which he was pre-scheduled for a follow-up with his specialist in 10 days. Despite previous negative results, it was also suspected that the pain could be related to pacemaker lead placement, migration, or perforation. Initial workup included complete blood cell count (CBC), comprehensive metabolic panel (CMP), prothrombin time test and international normalized ratio (PT/INR), lipase, troponin, thyroid-stimulating hormone (TSH), D-dimer, EKG (Figure 1), and CT chest, abdomen, and pelvis.

The CBC and CMP were unremarkable. Coagulation studies showed a PT/INR of 11.3/1.0 (reference range: 9.5-13.0 sec/0.8-1.2). Lipase was within normal limits at 74 U/L (reference range: 10-140 U/L). Troponin was mildly elevated at 17 ng/L (reference range: <14 ng/L), raising concern for possible myocardial injury; however, a repeat measurement downtrended to 16 ng/L, reducing suspicion. TSH was normal at 1.7 µIU/mL (reference range: 0.5-4.5). D-dimer was elevated at 855 ng/mL (reference range: <500 ng/mL), prompting further evaluation for pulmonary embolism (Table 1).

**Table 1:** Emergency department lab results (H): High; result is above the reference range. (L): Low; result is below the reference range. Blank: Result is within the normal reference range. ALT, alanine aminotransferase; AST, aspartate aminotransferase; BUN, blood urea nitrogen; eGFR, estimated glomerular filtration rate; FEU, fibrinogen-equivalent unit; INR, international normalized ratio; MCV, mean corpuscular volume; P, plasma; RBC, red blood cell; S, serum; WBC, white blood cell

Test	Reference range and units	Result
Hemoglobin	13.2-16.6 g/dL	14.9
Hematocrit	38.3-48.6%	47.7
Erythrocytes	4.35-5.65 x10¹²/L	4.88
MCV	78.2-97.9 fL	97.7
RBC distribution width	11.8-14.5%	14.2
Platelet count	135-317 x10⁹/L	249
Leukocytes	3.4-9.6 x10⁹/L	9.4
Neutrophils	1.56-6.45 x10⁹/L	5.58
Lymphocytes	0.95-3.07 x10⁹/L	1.64
Monocytes	0.26-0.81 x10⁹/L	0.96 (H)
Eosinophils	0.03-0.48 x10⁹/L	1.11 (H)
Basophils	0.01-0.08 x10⁹/L	0.14 (H)
Nucleated RBC	/100 WBC	0.0
Erythrocyte sedimentation rate	0-22 mm/1 h	24 (H)
Prothrombin time, P	9.4-12.5 sec	11.3
INR	0.9-1.1	1.0
D-dimer, P	≤500 ng/mL FEU	855 (H)
Sodium, S	135-145 mmol/L	140
Potassium, S	3.6-5.2 mmol/L	4.2
Chloride, S	98-107 mmol/L	102
Bicarbonate, S	22-29 mmol/L	27
Anion gap	7-15	11
BUN, S	8.0-24.0 mg/dL	13.0
Creatinine	0.74-1.35 mg/dL	1.11
eGFR	≥60 mL/min/BSA	68
Calcium, total, S	8.8-10.2 mg/dL	9.8
Glucose, S	70-140 mg/dL	163 (H)
Magnesium	1.7-2.3 mg/dL	2.2
Bilirubin, total, S	0.0-1.2 mg/dL	1.2
Bilirubin, direct, S	0.0-0.3 mg/dL	0.3
ALT, S	7-55 U/L	14
AST, S	8 - 48 U/L	20
Alkaline phosphatase, S	40-129 U/L	88
Protein, total, S	6.3 - 7.9 g/dL	7.9
Albumin, S	3.5-5.0 g/dL	4.5
Lipase, S	13-60 U/L	74 (H)
Troponin, baseline (5th gen)	≤15 ng/L	17 (H)
Troponin, 2 h (5th gen)	≤15 ng/L	16 (H)

**Figure 2:**
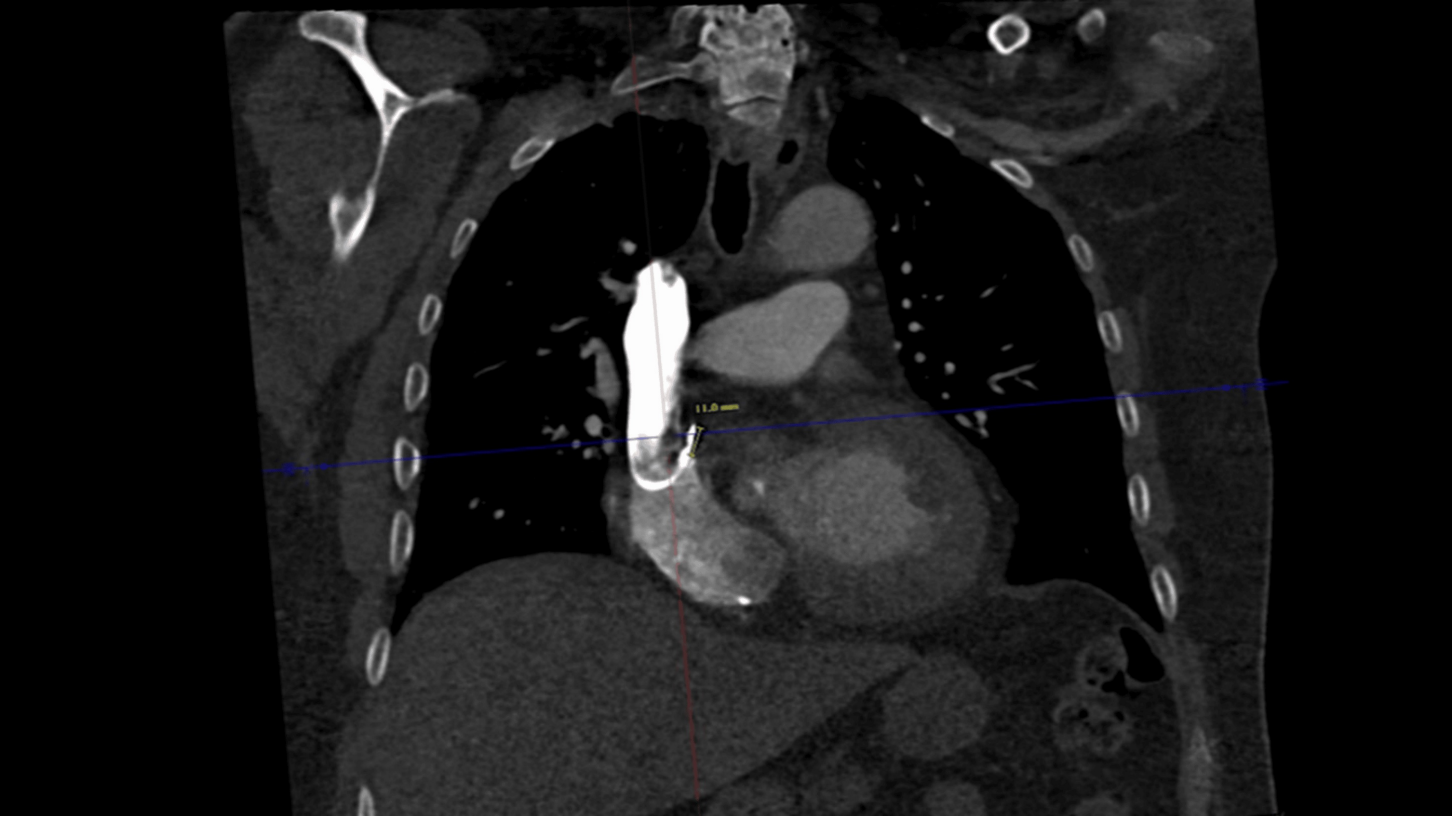
CT imaging of the chest revealed migration of the right atrial pacemaker lead. CT imaging of the chest revealed migration of the right atrial pacemaker lead into the medial portion of the proximal right atrium, positioned posterior to the aorta at the right atrial-superior vena cava junction.

**Figure 3:**
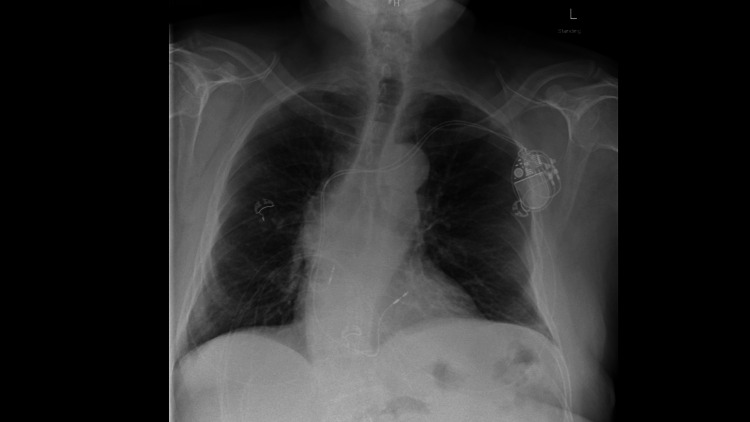
CXR demonstrated a U-shaped curvature of the proximal pacemaker lead at the cavoatrial junction. CXR demonstrated a U-shaped curvature of the proximal pacemaker lead at the cavoatrial junction. No focal consolidation, pleural effusion, or pneumothorax was identified.

CT imaging of the chest revealed migration of the right atrial pacemaker lead into the medial portion of the proximal right atrium, positioned posterior to the aorta at the right atrial-superior vena cava junction (Figure 2). CXR demonstrated a U-shaped curvature of the proximal pacemaker lead at the cavoatrial junction. No focal consolidation, pleural effusion, or pneumothorax was identified (Figure 3). CT of the abdomen and pelvis demonstrated mild, uncomplicated acute left colonic diverticulitis. Additionally, a stable 4.3 cm infrarenal abdominal aortic aneurysm and a 2.3 cm right common iliac artery aneurysm were noted, both unchanged from prior imaging.

He was admitted to the hospital under the cardiology service for further workup and evaluation of his pacemaker lead perforation and diverticulitis. A transthoracic echocardiogram revealed preserved left ventricular function with an ejection fraction of 61%, no regional wall motion abnormalities, and no pericardial effusion. Pacemaker interrogation showed stable lead sensing, impedance, and thresholds, all within normal limits, with no indication for lead revision. In the absence of hemodynamic instability and previous lead revision several months before, the cardiologist advised no immediate intervention and outpatient follow-up with his electrophysiologist, as repositioning the lead could decrease right atrial lead impedance and potentially induce pericardial effusion. He was treated with pantoprazole 40 mg daily for two weeks, with a follow-up scheduled with his primary care physician.

## Discussion

Myocardial perforation can be classified based on the time of occurrence relative to the device insertion. It is considered acute if it occurs within 24 hours, subacute if it happens between 1 day and 30 days, and chronic if it occurs more than 30 days after implantation [11]. While the overall incidence of myocardial perforation is reported to be between 1.7% and 7%, delayed lead perforation is considered to be exceptionally rare [10]. One study reported an overall lead perforation rate of 15% on CT scans, with atrial leads perforating more frequently (15%) than ventricular leads (6%). Ventricular implantable cardioverter-defibrillator leads also showed a higher perforation rate (14%) than ventricular pacemaker leads (3%) in this study; however, this particular study focused on asymptomatic perforations detected incidentally on CT [13].

Several factors can contribute to pacemaker lead perforation. Active fixation leads are more commonly associated with subacute perforation compared to passively fixed-tined leads [11]. The use of helical screw ventricular leads has also been identified as a predictor of post-implant effusion, a sign of perforation. Additionally, the concomitant use of a temporary transvenous pacemaker and steroid use within seven days before implant have been found to be strong predictors of symptomatic pericardial effusion after permanent pacemaker placement. Other weaker predictors include a low BMI (<20), older age, and longer fluoroscopy times. Conversely, a right ventricular systolic pressure ≥ 35 mmHg and a BMI ≥ 30 may be associated with a lower risk of perforation [5]. In the case of delayed perforation, the low profile of more recent leads with a decreased diameter might lead to increased force per unit area, potentially contributing to this complication even with flexible and looped leads, and technical factors during implantation also play a role [10].

 The clinical presentation of myocardial perforation can vary significantly. Patients may experience chest pain, which can be continuous or pleuritic, syncope, dyspnea, or even dizziness [9-11]. However, perforation can also be asymptomatic [13]. Right atrial lead perforation can irritate adjacent structures, including the diaphragm and phrenic nerve, resulting in referred or atypical pain patterns. Device interrogation may reveal non-specific findings such as decreased impedance, increased stimulation thresholds, or altered sensing, but normal pacemaker function does not exclude this diagnosis [13].

Chest radiography may show a loss of the typical lead shape, such as the italic S-shape of a ventricular lead [11]. Transthoracic echocardiography can visualize the penetration of the ventricular lead through the myocardium into the pericardium, potentially with or without pericardial effusion [11]. CT scan is considered superior to echocardiography and chest radiography for diagnosing cardiac perforation and is emerging as the gold standard. It can reveal the lead's trajectory and position relative to the myocardium and pericardium. A positive "epicardial fat-pad sign" on radiographic imaging, where a distance of less than 3 mm separates the lead tip from the epicardial fat stripe, can also suggest perforation. Point-of-care ultrasound in the ED can raise suspicion for lead perforation by revealing a pericardial effusion.

The management of pacemaker lead perforation depends on the clinical presentation, the presence and size of pericardial effusion, and the patient's hemodynamic status. In a reported case of subacute perforation, the patient was referred to surgery, where the lead was successfully reintegrated into the myocardium [10]. For recent implantations, transvenous lead extraction may be considered, with simple traction or specialized tools, with surgical backup in case of bleeding. In cases of delayed perforation, lead extraction under transesophageal echocardiographic observation with cardiac surgery backup is often recommended, even if the patient is hemodynamically stable [11]. Some advocate for extraction even in asymptomatic cases, while others suggest that extraction of a well-functioning, chronically perforated lead might not be necessary [13]. Surgical repositioning of the lead or other surgical interventions might be required in certain situations. Lead perforation can lead to serious complications, including pacemaker failure, hemopericardium, cardiac tamponade, pericarditis, pleural effusion, pneumothorax, and even death. Delayed perforation has been associated with chest pain, hemopneumothorax, and pneumothorax, but cardiac tamponade or death are less commonly reported in such cases compared to acute perforation [10].

## Conclusions

Pacemaker lead perforation is a notable complication with varying presentations and timelines. Understanding risk factors, potential symptoms, and appropriate diagnostic and management strategies is crucial for clinicians caring for patients with implanted cardiac devices. Advanced imaging techniques such as CT scans are increasingly important in diagnosing, especially in subacute and delayed presentations. The management approach must be tailored to the patient's clinical condition.
